# Research on the aging-suitability of community outdoor spaces in Zhengzhou based on the analytic hierarchy process and semantic differential method

**DOI:** 10.3389/fpubh.2024.1452120

**Published:** 2024-10-18

**Authors:** Chang Gao, Shengnan Wang, Pengpeng Li, Wenbo Jia, Kai Feng

**Affiliations:** ^1^Research Affairs Office, Henan University of Animal Husbandry and Economy, Zhengzhou, China; ^2^Mental Health Service Center, Henan University of Animal Husbandry and Economy, Zhengzhou, China; ^3^School of Horticulture, Hunan Agricultural University, Changsha, China; ^4^School of Management, Zhengzhou University, Zhengzhou, China; ^5^Academic Affairs Office, Henan University of Animal Husbandry and Economy, Zhengzhou, China

**Keywords:** aging-suitability, community outdoor spaces, outdoor spaces, analytic hierarchy process, semantic differential method

## Abstract

**Background:**

As global populations age, the suitability of community outdoor spaces for older adult residents has become a critical urban planning concern. However, comprehensive evaluation methods for assessing these spaces are lacking. Objective: This study aims to evaluate the aging-suitability of community outdoor spaces in Zhengzhou, China, and provide optimization suggestions for urban planners and policymakers.

**Methods:**

An evaluation index system was established using the analytic hierarchy process (AHP), with aging-suitability as the goal layer. The criteria layer included outdoor behavioral activities, green space configuration, transportation and road systems, and basic service facilities. The semantic differential (SD) method was employed to conduct a comprehensive evaluation among older adult residents in three communities (A, B, and C) in Zhengzhou city.

**Results:**

The evaluation revealed that older adult residents generally had a favorable assessment of their community spaces’ aging-suitability. Community A received the highest overall rating. Green space configuration and transportation systems emerged as critical factors influencing aging-suitability.

**Conclusion:**

The combined AHP-SD approach proved effective in evaluating the aging-suitability of community outdoor spaces. The study identified key areas for improvement in each community, with variations in strengths and weaknesses across the three sites. Implications: Based on the findings, optimization suggestions are proposed in four aspects: enhancing outdoor behavioral activities, improving green space landscapes, refining road transportation systems, and upgrading basic service facilities. These recommendations can guide urban planners and policymakers in creating more age-friendly community environments.

## 1 Introduction

According to the classification criteria established by the United Nations in 1956 regarding “The Aging of Population and its Socio-Economic Consequences,” a country or region is considered to be entering an aging phase when the proportion of the population aged 65 and above exceeds 7%. The 1982 Vienna International Plan of Action on Aging determined that a proportion exceeding 10% signifies severe aging. Currently, the global population is exhibiting a trend towards aging. As per World Health Organization statistics, at the beginning of the 21st century, the global older adult population aged 60 and above reached 620 million, accounting for 10% of the total population (1). From 2020 to 2030, the population over 60 is expected to increase from 1 billion to 1.4 billion, with the older adult population’s proportion growing by 34%, surpassing one-sixth of the world’s population (1–3).

This global aging trend has significant implications for urban planning and public health, particularly concerning the suitability of community outdoor spaces for older adult residents (4). Recent studies have highlighted the importance of age-friendly environments in promoting active aging. For instance, (39) found that well-designed outdoor spaces can encourage physical activity among older adults, while Sugiyama and Thompson (40) emphasized the role of accessible green spaces in enhancing mental well-being. However, there is a gap in the literature regarding comprehensive evaluation methods for assessing the aging-suitability of community outdoor spaces, particularly in rapidly urbanizing contexts (5–8).

In China, most older adult individuals still prefer the “family” and “community” care models (9, 10). This phenomenon can be attributed to two main factors: firstly, the older adult have developed a dependence on their long-term living environments; secondly, since most of their savings during their younger years have been invested in their current housing, they lack sufficient financial capacity to purchase new residences (11, 12). Currently, the facilities in most communities where the older adult reside are aging, with outdated and insufficient equipment, limited green space, and a general lack of accessible pathways (13). As they age, the older adult’s physical functions gradually decline, and their demands for community environment quality correspondingly increase (11). Their range of outdoor activities significantly reduces, usually confined within the community, such as in green spaces, plazas, and building entrances, for leisure and entertainment activities (9, 14–16).

Given these challenges, this study aims to address the following research questions: How can the aging-suitability of community outdoor spaces be effectively evaluated? What are the key factors influencing the aging-suitability of community outdoor spaces? How do different communities compare in terms of aging-suitability, and what improvements can be suggested? To answer these questions, we propose an innovative approach combining the analytic hierarchy process (AHP) and semantic differential (SD) method. This mixed-method approach allows for both objective weighting of factors and subjective evaluation by older adult residents, providing a more comprehensive assessment than previous studies.

Zhengzhou, the capital of Henan Province located in central China, covers an area of 7,567 square kilometers and has a permanent population of approximately 12.6 million, with an urbanization rate of 79.4% (17–19, 41). According to the seventh national population census, as of November 1, 2020, the population aged 60 and above in Zhengzhou was 1,617,392, accounting for 12.84%, and those aged 65 and above were 1,130,977, accounting for 8.98% (42). Compared to the sixth national population census in 2010, the proportion of the population aged 60 and above increased by 2.17 percentage points, and those aged 65 and above increased by 1.82 percentage points (43).

We chose Zhengzhou as our case study for several reasons. First, it exemplifies the rapid urbanization and aging trends observed in many Chinese cities. Second, as a second-tier city, Zhengzhou’s experience can provide insights applicable to many similar urban areas in China and potentially other developing countries facing similar demographic shifts. By focusing on the aging-suitability of outdoor spaces in Zhengzhou, this study contributes to the growing body of literature on age-friendly urban design, offering a novel evaluation framework that can be applied and adapted in various urban contexts.

Therefore, conducting evaluative research on the aging-suitability of community outdoor spaces in Zhengzhou is of significant importance to public health. It not only helps to reduce the risk of accidental injuries among the older adult but also encourages them to actively participate in outdoor activities, enhancing their physical fitness and psychological health. This, in turn, improves the overall quality of life and health level of the older adult population, contributing to the perfection and development of the public health system. Additionally, the research results can provide a basis for the government and relevant departments to formulate and adjust older adult care service policies, ensuring that the policies are more scientific and reasonable.

## 2 Materials and methods

### 2.1 Research Design

In accordance with the principles of the analytic hierarchy process (AHP), the evaluation system for the aging-suitability of community outdoor spaces was established. This involved an analysis of the interrelationships among various factors within the system and the construction of a hierarchical structure. Subsequently, experts from fields such as public health, landscape architecture, community services, psychological health, and nursing were invited to perform pairwise comparisons of the importance of criteria at each level, forming a judgment matrix. The relative weights of the elements being compared for each criterion were calculated from the judgment matrix, followed by a consistency check. Finally, the overall ranking weights for each level in relation to the system were computed and ordered.

Building on this foundation, the semantic differential (SD) method was employed to conduct an in-depth survey study of three representative communities within Zhengzhou city. These communities were selected based on the following principles: (1) Geographic diversity within Zhengzhou; (2) Variety in community types (established and newer developments); (3) Accessibility and significant older adult population.

The selected communities were: (1) Community A: Located on Longzihu Street in the Jinshui District of Zhengzhou, Henan Province, representing an established urban neighborhood. (2) Community B: In Caicheng Town of the Zhengdong New District in the Jinshui District of Zhengzhou, exemplifying a newer, planned community. (3) Community C: On Zhengguang Road in the Jinshui District of Zhengzhou, chosen as a mixed-age community with recent renovations.

Through field research and questionnaire surveys, the aging-suitability of community outdoor spaces was assessed. The survey questionnaires were distributed to residents aged 60 and above within these three communities, with a total of 210 questionnaires issued. Out of these, 191 were retrieved, and 183 were deemed valid. Specifically, 62 valid questionnaires were collected from Community A, 68 from Community B, and 53 from Community C.

In addition to the surveys, in-depth interviews were conducted with a subset of respondents. These interviews covered topics such as daily use patterns of outdoor spaces, perceived benefits and challenges, and suggestions for improvements. Interviewees were selected to ensure diversity in age (60–85 years), gender, education level, and physical ability.

### 2.2 Data source

The study constructs an evaluation system for the aging-suitability of community outdoor spaces through the analytic hierarchy process (AHP), based on literature review and expert consultation (20–22). Subsequently, a comprehensive analysis is conducted using the semantic differential (SD) method (23), combined with a questionnaire survey targeting the older adult residents of the community. This approach integrates both quantitative and qualitative evaluation methods to analyze the aging-suitability of community outdoor spaces.

The AHP decomposes the problem into different constituent factors according to the nature of the problem and the overall goal to be achieved. These factors are then aggregated and combined at different levels according to their interrelated influences and hierarchical relationships, forming a multi-level analytical structure model. This ultimately reduces the problem to the relative importance of the lowest level (options, measures, etc.) in relation to the highest level (overall goal).

The “semantic differential method,” a post-evaluation technique originating from psychological assessment experiments, was proposed by Osgood (23). It involves measuring the psychological perceptions of respondents through verbal scales and then quantitatively describing the concepts and constructs of the research subject through the analysis of established scales. In this paper, the SD method is applied to evaluate the aging-suitability of community outdoor spaces in Zhengzhou, effectively reflecting the satisfaction levels of the older adult community group.

## 3 Determination of indicators and weights by analytic hierarchy process

In this study, the analytic hierarchy process (AHP) was employed to determine the weights of the evaluation indicators for assessing the aging-suitability of community outdoor spaces. AHP is a structured decision-making method that allows for the quantification of subjective assessments of relative importance among a set of criteria (21). The process involves decomposing a complex problem into a hierarchical structure and then making pairwise comparisons to derive quantitative weights.

### 3.1 Establishment of the hierarchical structure

Based on the literature review and field surveys, a hierarchical structure model was established for the evaluation of aging-suitability of community outdoor spaces. The hierarchy consists of three levels:

- Goal level: Evaluation of the aging-suitability of community outdoor spaces.
- Criteria level: Four key elements were identified as criteria:

1. Outdoor behavioral activities: Encompasses factors that promote social interaction and physical activity among the older adult.
2. Green space landscape: Includes aspects related to the aesthetic and functional qualities of green spaces that benefit the older adult.
3. Road traffic system: Pertains to the safety, accessibility, and convenience of the transportation and road infrastructure within the community.
4. Basic service facilities: Covers the availability and adequacy of facilities that support the daily needs and well-being of the older adult.

- Sub-criteria level: Each criterion was further subdivided into specific indicator factors, resulting in a total of 20 sub-criteria. These sub-criteria were identified to capture detailed aspects influencing the aging-suitability of community outdoor spaces.

### 3.2 Construction of pairwise comparison matrices

To determine the relative importance of the criteria and sub-criteria, pairwise comparison matrices were constructed following the standard AHP methodology. A panel of 15 experts was assembled, comprising professionals in public health, landscape architecture, urban planning, gerontology, and community services. The experts were invited to provide their judgments on the relative importance of each pair of criteria and sub-criteria.

Using Saaty’s scale of relative importance (ranging from 1 to 9), experts rated each pair of elements by considering which element is more important and by how much. The individual judgments were then aggregated using the geometric mean method to form the final pairwise comparison matrices.

### 3.3 Calculation of weights and consistency check

The pairwise comparison matrices were used to compute the priority vectors (weights) through the eigenvalue method. The principal eigenvector of each matrix was calculated to derive the weights of the criteria and sub-criteria.

To ensure the reliability of the judgments, the consistency ratio (CR) was calculated for each matrix using the formula: CR = CI/RI, where CI is the consistency index, and RI is the random index corresponding to the size of the matrix. A CR value less than 0.1 indicates acceptable consistency in the judgments. All the matrices in this study had CR values below 0.1, confirming the consistency of the expert evaluations.

### 3.4 Results of weight calculation

**Figure 1:**
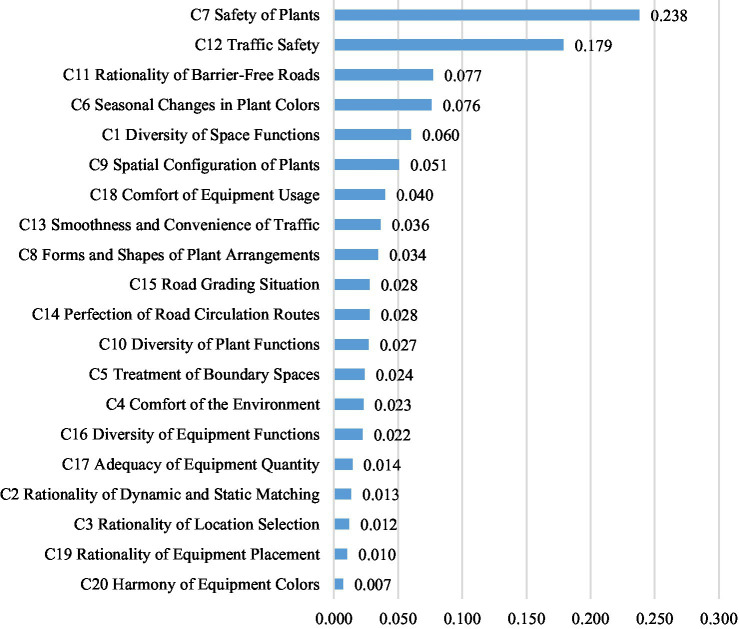
Comprehensive weight value ranking.

The calculated weights for the criteria and sub-criteria are presented in Table 1, with a visual representation in Figure 1. The composite weight values were obtained by multiplying the weights of the criteria and sub-criteria through the hierarchy, providing a quantitative measure of the relative importance of each indicator in the overall evaluation.

**Table 1:** Hierarchical structure of evaluation indicators with overall ranking and weight values.

Goal level	Criteria level	Criteria weight value	Scheme level	Scheme weight	Composite weight value
Evaluation of aging-suitability of community outdoor spaces	B1 outdoor behavioral activities	0.132	C1 diversity of space functions	0.224	0.030
			C2 rationality of dynamic and static matching	0.116	0.015
			C3 rationality of location selection	0.145	0.019
			C4 comfort of the environment	0.438	0.058
			C5 treatment of boundary spaces	0.077	0.010
	B2 green space landscape	0.426	C6 seasonal changes in plant colors	0.179	0.076
			C7 safety of plants	0.559	0.238
			C8 forms and shapes of plant arrangements	0.080	0.034
			C9 spatial configuration of plants	0.119	0.051
			C10 diversity of plant functions	0.063	0.027
	B3 road traffic system	0.348	C11 rationality of barrier-free roads	0.222	0.077
			C12 traffic safety	0.515	0.179
			C13 smoothness and convenience of traffic	0.105	0.036
			C14 perfection of road circulation routes	0.079	0.028
			C15 road grading situation	0.079	0.028
	B4 basic service equipment	0.094	C16 diversity of equipment functions	0.238	0.022
			C17 adequacy of equipment quantity	0.153	0.014
			C18 comfort of equipment usage	0.424	0.040
			C19 rationality of equipment placement	0.110	0.010
			C20 harmony of equipment colors	0.075	0.007

The results indicate that among the criteria, green space landscape (weight: 0.426) and road traffic system (weight: 0.348) are of higher importance compared to outdoor behavioral activities (weight: 0.132) and basic service facilities (weight: 0.094). At the sub-criteria level, C7 safety of plants (composite weight: 0.238) and C12 traffic safety (composite weight: 0.179) emerged as the most significant factors.

## 4 Analysis of the aging-suitability evaluation for community outdoor spaces in Zhengzhou based on SD

### 4.1 Establishment of SD adjective pairs

Building upon the 20 evaluation factors established earlier, we collected related pairs of adjectives and conducted a standardized selection process. This allowed researchers to analyze the evaluation indicators more clearly and perform quantitative statistics. The selection principle for adjective pairs typically involves choosing those with diametrically opposite meanings and excluding those with ambiguous opposites. Furthermore, each pair of adjectives should have a relatively balanced evaluation scale, with any overly biased pairs being discarded. In the application of the Semantic Differential (SD) method, adjective pairs that are frequently used and align with the aging-suitability of community outdoor spaces were selected, culminating in a predefined set of 20 adjective pairs, as shown in Table 2.

**Table 2:** Aging-suitability SD evaluation factors and adjective pairs.

Evaluation factor	Description of evaluation factor	Adjective pair
C1 diversity of space functions	Whether the space functions are diverse	Monofunctional—multifunctional
C2 rationality of dynamic and static matching	Whether the dynamic and static matching is reasonable	Chaotic—orderly
C3 rationality of location selection	Whether the location selection is reasonable	Irrational—rational
C4 comfort of the environment	Whether the community environment is comfortable	Uncomfortable—comfortable
C5 treatment of boundary spaces	Whether the treatment of boundary spaces is appropriate	Inappropriate—appropriate
C6 seasonal changes in plant colors	Whether the seasonal color changes of plants are harmonious	Monotonous—harmonious
C7 safety of plants	Whether the plants pose any safety hazards	Dangerous—safe
C8 forms and shapes of plant arrangements	Whether the plant arrangements cater to the older adult	Contrary—catering
C9 spatial configuration of plants	Whether the spatial configuration of plants is suitable	Unsuitable—suitable
C10 diversity of plant functions	Whether the functions of plants are diverse and rich	Monofunctional—rich in functions
C11 rationality of barrier-free roads	Whether the barrier-free road settings are reasonable	Irrational—rational
C12 traffic safety	Whether the community traffic safety meets standards	Dangerous—safe
C13 smoothness and convenience of traffic	Whether the community traffic is smooth and convenient	Cumbersome—convenient
C14 perfection of road circulation routes	Whether the road circulation routes are well-established	Lacking—well-established
C15 road grading situation	Whether the road branching situation is reasonable	Irrational—rational
C16 diversity of equipment functions	Whether the equipment functions are diverse	Single—diverse
C17 adequacy of equipment quantity	Whether the quantity of equipment is sufficient	Lacking—sufficient
C18 comfort of equipment usage	Whether the use of equipment is comfortable for the older adult	Uncomfortable—comfortable
C19 rationality of equipment placement	Whether the placement of equipment is reasonable	Mismatched—matched
C20 harmony of equipment colors	Whether the colors of the equipment are harmonious	Discordant—harmonious

### 4.2 SD results

The previously determined 20 indicators were used as evaluation items to assess the aging-suitability of community outdoor spaces by the older adult within the surveyed communities. After consulting relevant literature and expert opinions, the evaluation scale was divided into five levels, assigned scores from 1 to 5 in ascending order. The corresponding evaluation levels were set as “Very Poor,” “Poor,” “Fair,” “Good,” and “Very Good.” This led to the creation of a satisfaction survey questionnaire for the aging-suitability of community outdoor spaces in Zhengzhou.

The survey was distributed among the older adult residents, all aged 60 and above, in three communities, requesting their assistance in completion and collection. A total of 210 questionnaires were distributed, 191 were retrieved, and after discarding a few improperly filled ones, 183 valid questionnaires remained. Specifically, 62 valid questionnaires were collected from Community A, 68 from Community B, and 53 from Community C. After compiling the survey data from each community, the results were entered into Excel software to calculate a comprehensive evaluation for each age-friendliness indicator of community outdoor spaces. The comprehensive evaluation data were then quantified and plotted in Table 3.

**Table 3:** SD comprehensive evaluation table.

Rank	Community A (*N* = 62)		Community B (*N* = 68)		Community C (*N* = 53)	
	Evaluation factor	Mean score	Evaluation factor	Mean score	Evaluation factor	Mean score
1	C9 spatial configuration of plants	3.887	C7 safety of plants	3.544	C13 smoothness and convenience of traffic	3.887
2	C13 smoothness and convenience of traffic	3.710	C13 smoothness and convenience of traffic	3.500	C12 traffic safety	3.623
3	C12 traffic safety	3.694	C14 perfection of road circulation routes	3.397	C17 adequacy of equipment quantity	3.321
4	C14 perfection of road circulation routes	3.661	C12 traffic safety	3.353	C15 road grading situation	3.189
5	C1 diversity of space functions	3.661	C1 diversity of space functions	3.338	C16 diversity of equipment functions	3.189
6	C7 safety of plants	3.645	C2 rationality of dynamic and static matching	3.294	C11 rationality of barrier-free roads	3.170
7	C15 road grading situation	3.629	C3 rationality of location selection	3.294	C14 perfection of road circulation routes	3.151
8	C16 diversity of equipment functions	3.532	C15 road grading situation	3.221	C7 safety of plants	3.132
9	C18 comfort of equipment usage	3.500	C4 comfort of the environment	3.206	C9 spatial configuration of plants	3.113
10	C17 adequacy of equipment quantity	3.468	C5 treatment of boundary spaces	3.206	C4 comfort of the environment	3.075
11	C11 rationality of barrier-free roads	3.419	C18 comfort of equipment usage	3.191	C5 treatment of boundary spaces	3.075
12	C3 rationality of location selection	3.403	C11 rationality of barrier-free roads	3.132	C6 seasonal changes in plant colors	3.057
13	C20 harmony of equipment colors	3.403	C9 spatial configuration of plants	3.044	C18 comfort of equipment usage	3.038
14	C4 comfort of the environment	3.387	C6 seasonal changes in plant colors	3.000	C3 rationality of location selection	3.019
15	C2 rationality of dynamic and static matching	3.323	C10 diversity of plant functions	2.956	C8 forms and shapes of plant arrangements	3.000
16	C19 rationality of equipment placement	3.323	C16 diversity of equipment functions	2.897	C20 harmony of equipment colors	2.943
17	C5 treatment of boundary spaces	3.210	C19 rationality of equipment placement	2.853	C2 rationality of dynamic and static matching	2.925
18	C6 seasonal changes in plant colors	3.145	C20 harmony of equipment colors	2.838	C1 diversity of space functions	2.906
19	C10 diversity of plant functions	2.774	C8 forms and shapes of plant arrangements	2.691	C19 rationality of equipment placement	2.887
20	C8 forms and shapes of plant arrangements	2.387	C17 adequacy of equipment quantity	2.309	C10 diversity of plant functions	2.792

### 4.3 SD curve

**Figure 2:**
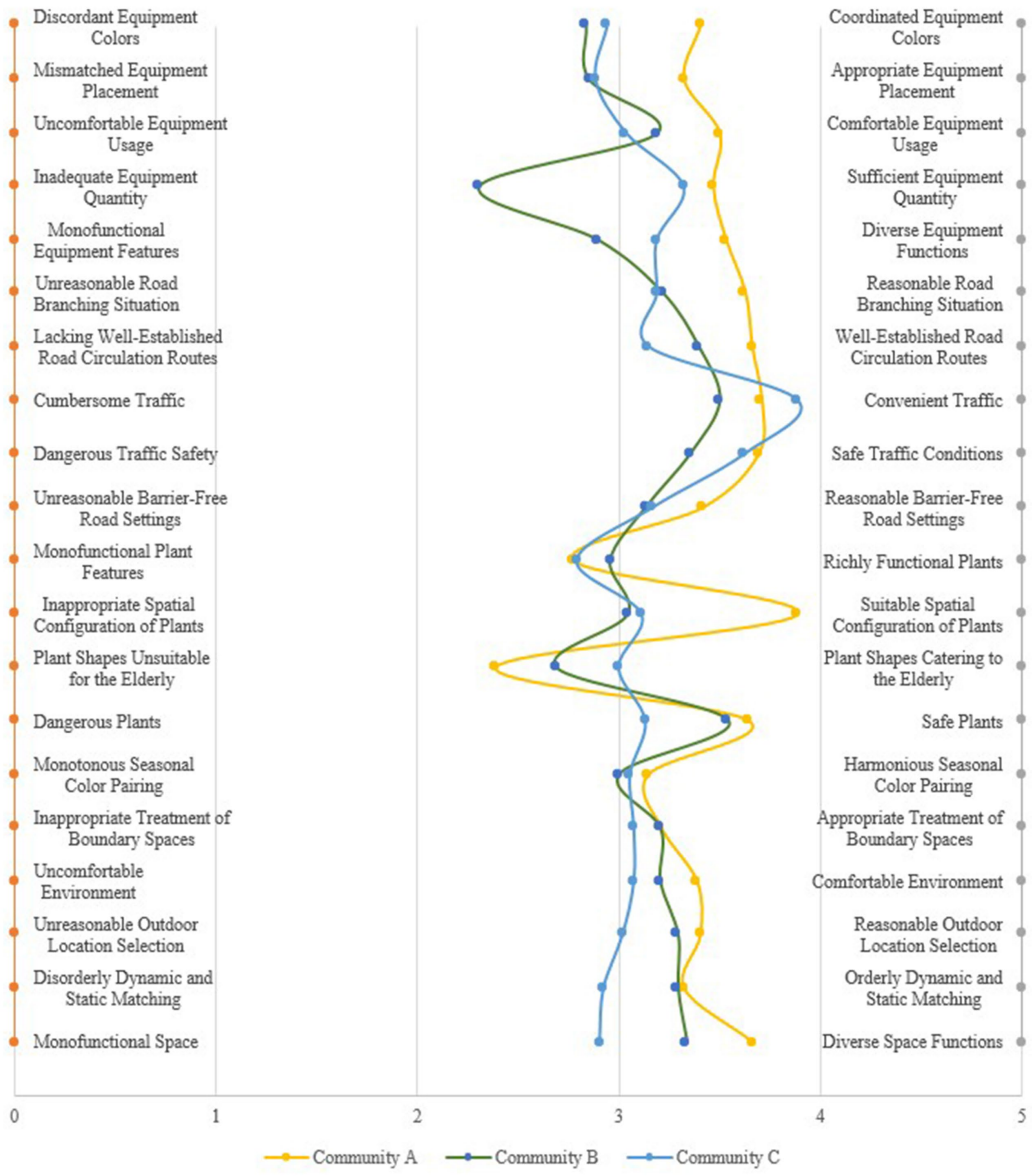
SD comprehensive evaluation curve of three communities.

The comprehensive evaluation scores of the 20 indicator factors from the aforementioned Communities A, B, and C were imported into Excel. Subsequently, by combining the average scores of the adjective pairs for each evaluation indicator, an SD curve comprehensive evaluation chart was created, as illustrated in Figure 2.

Analysis of the data distribution in the aforementioned charts indicates that Community A’s scores predominantly fall within the range greater than 3 but less than or equal to 4, with significant curvature occurring in the aspect of green space configuration, resulting in relatively high overall scores. Community B exhibits commendable performance in terms of traffic fluidity and plant safety, yet scores the lowest in equipment quantity sufficiency, with evaluative factors largely distributed in the range less than 3 but greater than or equal to 2. The semantic differential (SD) curve for Community C is comparatively even, scoring highest among the three communities in traffic fluidity and convenience. However, it ranks lowest in the diversity of plant functions.

### 4.4 Comprehensive evaluation results considering weights

This study employs a combined approach of analytic hierarchy process (AHP) and semantic differential (SD) analysis to conduct an in-depth evaluation of aging-suitability indicators within communities A, B, and C. Initially, the AHP method was utilized to ascertain the relative importance of aging-suitability indicators within these communities. Subsequently, the SD method was applied to assess the satisfaction levels of the older adult regarding each evaluation factor across the three communities. The integration of these methods not only overcomes the limitations of quantification inherent in using AHP alone but also addresses the potential for case-specific issues that may arise from sole reliance on SD analysis, thereby rendering the research findings more scientific and comprehensive. After assigning values to the evaluation factors of each community using the semantic differential (SD) method, the average assigned value for each factor was obtained. By combining these with the comprehensive weight values of each evaluation index derived from the analytic hierarchy process (AHP), multiplying them together and summing up, the final comprehensive score for each community was calculated (see Table 4).

**Table 4:** Comprehensive evaluation results considering weights.

Evaluation factor	Composite weight value	Community A		Community B		Community C	
		Mean score	Results considering weights	Mean score	Results considering weights	Mean score	Results considering weights
C1 diversity of space functions	0.030	3.661	3.499	3.338	3.253	2.906	3.209
C2 rationality of dynamic and static matching	0.015	3.323		3.294		2.925	
C3 rationality of location selection	0.019	3.403		3.294		3.019	
C4 comfort of the environment	0.058	3.387		3.206		3.075	
C5 treatment of boundary spaces	0.010	3.210		3.206		3.075	
C6 seasonal changes in plant colors	0.076	3.145		3.000		3.057	
C7 safety of plants	0.238	3.645		3.544		3.132	
C8 forms and shapes of plant arrangements	0.034	2.387		2.691		3.000	
C9 spatial configuration of plants	0.051	3.887		3.044		3.113	
C10 diversity of plant functions	0.027	2.774		2.956		2.792	
C11 rationality of barrier-free roads	0.077	3.419		3.132		3.17	
C12 traffic safety	0.179	3.694		3.353		3.623	
C13 smoothness and convenience of traffic	0.036	3.7100		3.500		3.887	
C14 perfection of road circulation routes	0.028	3.661		3.397		3.151	
C15 road grading situation	0.028	3.629		3.221		3.189	
C16 diversity of equipment functions	0.022	3.532		2.897		3.189	
C17 adequacy of equipment quantity	0.014	3.468		2.309		3.321	
C18 comfort of equipment usage	0.040	3.500		3.191		3.038	
C19 rationality of equipment placement	0.010	3.323		2.853		2.887	
C20 harmony of equipment colors	0.007	3.403		2.838		2.943	

## 5 Discussion

This study presents a comprehensive evaluation of the aging-suitability of community outdoor spaces in Zhengzhou City, employing a combined analytic hierarchy process (AHP) and semantic differential (SD) methodological approach. The findings contribute to the growing body of research on urban environments that cater to an aging demographic, offering insights that can inform policy and design decisions.

The results indicate a clear hierarchy of importance among the criteria for aging-suitability, with green space landscape and road traffic system emerging as the most critical factors (24). This underscores the dual need for aesthetically pleasing environments that also prioritize safety and accessibility (25). Community A’s high overall score reflects its success in integrating these elements, suggesting that a well-rounded approach to community design can significantly enhance the user experience for the older adult (26, 27).

Our findings resonate with existing literature that emphasizes the importance of green spaces (28) and accessible transportation (29–31) for the well-being of older adults. However, the study also highlights areas where current practices fall short, such as the adequacy of equipment quantity in Community B, which contrasts with research advocating for ample and varied amenities in senior-friendly environments (32).

Community C, although performing adequately in traffic fluidity, demonstrates deficiencies in botanical function diversity, suggesting room for improvement through refined landscaping initiatives (33). The pragmatic implications of this research are multifaceted (34). Decision-makers and urban designers are advised to heed the identified areas for enhancement to foster more inclusive and senior-friendly environments. This encompasses the enrichment of greenery, augmentation of traffic systems, and assurance of basic service facility accessibility.

The study’s findings have practical implications for urban planners and policymakers. It suggests that investments in improving road traffic systems and green space landscapes can significantly enhance the aging-suitability of communities. Moreover, the need for sufficient and diverse equipment in community spaces highlights the importance of continuous assessment and updating of community facilities to meet the evolving needs of the older adult (35, 36).

Based on the findings, several policy recommendations can be proposed. Firstly, there should be a strategic allocation of resources towards the development and maintenance of green spaces that offer year-round visual appeal and safety. Secondly, road infrastructure should be designed with universal design principles to accommodate the mobility needs of the older adult. Thirdly, community equipment should be regularly reviewed and upgraded to ensure it meets the comfort and functionality requirements of older users (33, 37, 38).

In conclusion, this study underscores the critical role of community outdoor spaces in supporting the quality of life of the older adult. By identifying key areas for improvement and employing a robust evaluation methodology, the research provides a solid foundation for evidence-based urban planning and policy development. It is hoped that the findings will encourage further research and stimulate action towards creating more inclusive and aging-suitable urban environments.

## 6 Limitations and future research

While this study offers valuable insights into the design of aging-friendly community spaces, it is important to recognize its limitations. The sample size, limited to three communities within Zhengzhou, may not capture the full spectrum of diversity found in different urban environments. Future research should strive to include a wider array of communities, varying in size, location, and demographic composition, to ensure broader applicability of the results. Additionally, longitudinal studies would be beneficial to understand the dynamic nature of aging-suitability and its sustained impact on the older adult’s social engagement and health outcomes over time. Expanding the demographic and geographic scope in subsequent studies will help corroborate these initial findings and provide a more comprehensive understanding of the factors that contribute to making community spaces welcoming for the aging population.

## Data Availability

The raw data supporting the conclusions of this article will be made available by the authors, without undue reservation.
